# Molecular epidemiology and emergence of worldwide epidemic clones of *Neisseria meningitidis *in Taiwan

**DOI:** 10.1186/1471-2334-6-25

**Published:** 2006-02-15

**Authors:** Chien-Shun Chiou, Jui-Cheng Liao, Tsai-Ling Liao, Chun-Chin Li, Chen-Ying Chou, Hsiu-Li Chang, Shu-Man Yao, Yeong-Sheng Lee

**Affiliations:** 1 The Third Branch Office, Center for Disease Control, Taichung 408, Taiwan; 2 The Laboratory Research and Development, Center for Disease Control, Taipei 115, Taiwan; 3 The Sixth Branch Office, Center for Disease Control, Hualien 970, Taiwan

## Abstract

**Background:**

Meningococcal disease is infrequently found in Taiwan, a country with 23 million people. Between 1996 and 2002, 17 to 81 clinical cases of the disease were reported annually. Reported cases dramatically increased in 2001–2002. Our record shows that only serogroup B and W135 meningococci have been isolated from patients with meningococcal disease until 2000. However, serogroup A, C and Y meningococci were detected for the first time in 2001 and continued to cause disease through 2002. Most of serogroup Y meningococcus infections localized in Central Taiwan in 2001, indicating that a small-scale outbreak of meningococcal disease had occurred. The occurrence of a meningococcal disease outbreak and the emergence of new meningococcal strains are of public health concern.

**Methods:**

*Neisseria meningitidis *isolates from patients with meningococcal disease from 1996 to 2002 were collected and characterized by serogrouping, pulsed-field gel electrophoresis (PFGE) and multilocus sequence typing (MLST). The genetic relatedness and clonal relationship between the isolates were analyzed by using the PFGE patterns and the allelic profiles of the sequence types (STs).

**Results:**

Serogroups A, B, C, W135, Y, and non-serogroupable *Neisseria meningitidis *were, respectively, responsible for 2%, 50%, 2%, 35%, 9%, and 2% of 158 culture-confirmed cases of meningococcal disease in 1996–2002. Among 100 *N. meningitidis *isolates available for PFGE and MLST analyses, 51 different PFGE patterns and 30 STs were identified with discriminatory indices of 0.95 and 0.87, respectively. Of the 30 STs, 21 were newly identified and of which 19 were found in serogroup B isolates. A total of 40 PFGE patterns were identified in 52 serogroup B isolates with the patterns distributed over several distinct clusters. In contrast, the isolates within each of the serogroups A, C, W135, and Y shared high levels of PFGE pattern similarity. Analysis of the allelic profile of the 30 STs suggested the serogroup B isolates be assigned into 5 clonally related groups/ clonal complexes and 7 unique clones. The ST-41/44 complex/Lineage 3, and the ST-3439 and ST-3200 groups represented 79% of the serogroup B meningococci. In contrast, isolates within serogroups A, serogroup W135 (and C), and serogroup Y, respectively, simply belonged to ST-7, ST-11, and ST-23 clones.

**Conclusion:**

Our data suggested that serogroup B isolates were derived from several distinct lineages, most of which could either be indigenous or were introduced into Taiwan a long time ago. The serogroup A, W135 (and C), and Y isolates, respectively, belonged to the ST-7, ST-11, and ST-23, and the represented clones that are currently the major circulating clones in the world and are introduced into Taiwan more recently. The emergence of serogroup A, C and Y strains contributed partly to the increase in cases of meningococcal disease in 2001–2002.

## Background

*Neisseria meningitidis *is one of the major causative agents of bacterial meningitis and septicemia in children and young adults, with an estimated 500,000 cases and 50,000 deaths per year worldwide [1]. This organism is subdivided into 13 major serogroups based on the chemical and serological properties of the capsular polysaccharide [2]. Serogroups A, B, and C of this species have historically been the main cause of most endemics and epidemics of meningococcal disease [1]. However, serogroups W135 and Y meningococci have become more significant recently. Serogroup W135 meningococcus was responsible for the Hajj-associated meningococcal disease outbreak in 2000 and subsequent epidemics in Africa [3-5]. The incidence of serogroup Y meningococcal disease has also increased since the 1990s in North America [6-8].

Between 1996 and 2002, 17 to 81 clinical cases of meningococcal disease were reported annually in Taiwan, a country with 23 million people. Of 292 reported clinical cases, 158 were confirmed by bacterial culture. Our record shows that only serogroup B and W135 meningococci have been isolated from patients with meningococcal disease until 2000. However, serogroup A, C and Y meningococci were detected for the first time in 2001 and continued to cause disease through 2002. A few cases of the disease were caused by serogroup Y meningococcus, with most of infections localized in Central Taiwan in 2001, suggesting that a small-scale outbreak of meningococcal disease had occurred. The occurrence of a meningococcal disease outbreak and the emergence of new meningococcal strains are of public health concern.

Analyses of *N. meningitidis *isolates using molecular subtyping methods are useful for epidemiological studies. Among the subtyping techniques, pulsed-field gel electrophoresis (PFGE) and multilocus sequence typing (MLST) currently are the most widely adopted epidemiological tools for cluster designation, disease outbreak investigation, and tracking the global spread of meningococcal clones [9-11]. In this study, *N. meningitidis *isolates collected from patients in 1996–2002 were characterized using PFGE and MLST methods. The genotyping results are used to describe the molecular epidemiology of the recent meningococcal disease in Taiwan and infer the evolutionary origins of the causative agents.

## Methods

### Bacterial strains

A total of 158 *N. meningitidis *isolates were cultured from blood and cerebrospinal fluid specimens collected from patients with meningococcal disease in hospitals throughout Taiwan from 1996 to 2002. Bacterial isolates were sent to the four district laboratories of the Center for Disease Control, Taiwan, with reporting forms that contained patient information (name, sex, date of birth, residency, day of onset, and symptoms). Serogroup was determined at the four district laboratories using the slide agglutination method with antisera from Murex Biotech Ltd., Dartford, England [12]. There were 100 isolates available for genetic characterization. The PFGE size reference strain, *N. meningitidis *M413, was kindly provided by Dr. T. Popovic of the Centers for Disease Control and Prevention, Atlanta, Georgia, USA. The bacterial isolates were stored in 15% glycerol at -75°C until use.

### PFGE

The PFGE procedures described by Popovic et al. were applied in this study [9]. *Nhe*I-treated genomic DNA fragments of *N. meningitidis *M413 were used as the size reference markers. After electrophoresis, the gel was stained with 1 mg/L ethidium bromide and the PFGE profile recorded using a digital camera system (Kodak Electrophoresis Documentation and Analysis System 290) with 1792 × 1200 pixels.

### MLST

The primers for PCR amplification and sequencing of the housekeeping genes *abcZ *(putative ABC transporter), *adk *(adenylate kinase), *aroE *(shikimate dehydrogenase), *fumC *(fumarate hydratase), *gdh *(glucose-6-phosphate dehydrogenase), *pdhC *(pyruvate dehydrogenase subunit), *pgm *(phosphoglucomutase) were synthesized in Mission Biotech Corp. Taipei, Taiwan, according to the primer sequences of the *Neisseria *MLST database website [13] Bacterial DNA was extracted using a commercial kit (Viogene Blood & Tissue Genomic DNA Extraction Miniprep System, Taipei, Taiwan). The target DNA was amplified by PCR following the reaction conditions supplied at the above website. PCR amplified DNA was sent to Mission Biotech Corp. for nucleotide sequence determination. Sequence segments of the 7 genes (*abcZ*, 433 bp; *adk*, 465 bp; *aroE*, 490 pb; *fumC*, 465 bp; *gdh*, 501 bp; *pdhC*, 480 bp; *pgm*, 450 bp) from each isolate were compared with the previously observed allelic sequences in the *Neisseria *MLST database for identification of ST and the relationships of the STs with the defined clonal complex are automatically assigned. Sequences of new alleles were sent to the curator of the *Neisseria *MLST database for new allele and ST code assignment.

### Data analysis

The digital PFGE image was analyzed using BioNumerics software (Applied Maths, Maths, Kortrijik, Belgium). A PFGE pattern with one or more DNA bands different from the others was taken as a unique PFGE pattern and a new PFGE code was assigned. The discriminatory power of the two subtyping methods was evaluated by a discriminatory index as calculated by Hunter [14]. A dendrogram of PFGE patterns was constructed using the unweighted pair group with arithmetic averaging (UPGMA) method and the Dice similarity coefficient. Clonal relationships between the STs were constructed using the Minimum Spanning Tree (MST) method of the BioNumerics cluster analysis module. The MST method incorporated the BURST algorithm as a set of priority rules for cluster analysis [15].

## Results

During the period 1996 to 2002, 17 to 81 clinical cases of meningococcal disease were reported annually from hospitals to the Center of Disease Control, Taiwan. Each year between 11 and 46 cases were confirmed by the bacterial culturing method (Table 1). Serogroup B and W135 meningococci ranked as the first and second most common serogroups, responsible for 50% and 35% of the confirmed cases, respectively (Table 1). Only serogroups B and W135 meningococci were detected during 1996–2000. Serogroup A, C, and Y meningococci emerged for the first time in 2001 and continued to cause disease throughout 2002. Serogroup Y meningococcus caused more cases of meningococcal disease than serogroup A and C combined. Most of these cases were in Central Taiwan, suggesting that a small-scale outbreak of serogroup Y meningococcal disease apparently occurred at that time. The cases caused by the emerging serogroup A, C and Y strains as well as the increased cases caused by serogroup W135 were attributed to the dramatic increase in cases of meningococcal disease in 2001. Serogroup B strains also contributed to the increase of meningococcal disease in 2002.

**Table 1:** Reported and culture-confirmed cases of meningococcal disease, the prevalence of different serogroups of *Neisseria meningitidis *isolates in 1996–2002, and isolates available for genotyping in this study.

	No. case/isolate (No. genotyped)							Total
		
	1996	1997	1998	1999	2000	2001	2002	
Reported case	22	37	17	21	34	80	81	292
Confirmed^a^								
A						2 (1)	1 (1)	3 (2)
B	9 (6)	12 (7)	6 (3)	10 (6)	4	13 (12)	25 (18)	79 (52)
C						1 (1)	2 (2)	3 (3)
W135	4 (4)	7 (4)	4 (2)	1	9 (2)	19 (12)	11 (7)	55 (31)
Y						10 (8)	5 (3)	15 (11)
NG			2			1 (1)		3 (1)
Total	13 (10)	19 (11)	12 (5)	11 (6)	13 (2)	46 (35)	44 (31)	158 (100)

^a^NG: non-serogroupable.

**Figure 1:**
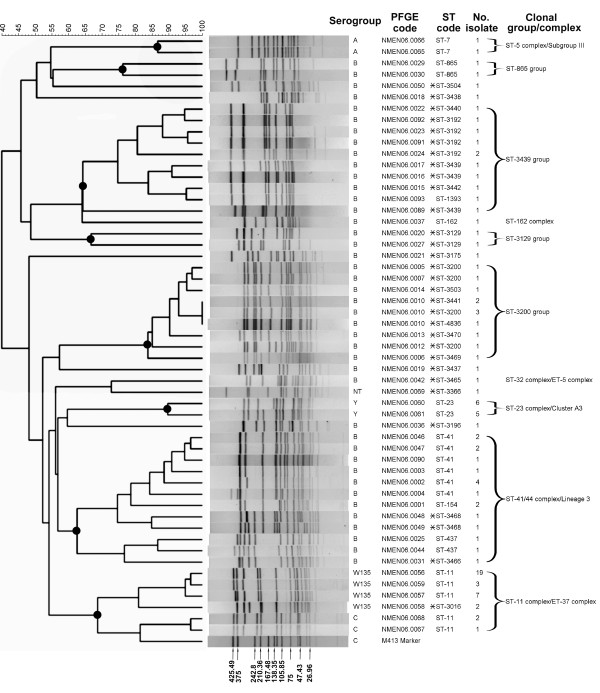
Dendrogram and PFGE patterns of *Nhe*I-digested chromosomal DNA of *Neisseria meningitidis *isolates and their association with serogroup, assigned PFGE code, ST code, and clonal group/complex. The dendrogram was constructed using BioNumerics software, with 2% optimization and 0.8% position tolerance, using the UPGMA algorithm and the Dice similarity coefficients. Dark circle (●) indicates a clonal group/complex. The sizes of the DNA bands from the marker strain (M413) are indicated in kb. Asterisk (*) indicates the ST sequence identified for the first time in this study.

Of the 158 *N. meningitidis *isolates, 100 were available for PFGE and MLST analyses. The analyses identified 51 different PFGE patterns and 30 STs. The indices of discriminatory power for PFGE and MLST were 0.95 and 0.87, respectively. Although the PFGE method exhibited higher discriminatory power than MLST, isolates with identical PFGE patterns (e.g. NMEN06.0010) were further differentiated into three STs (Figure 1).

The genetic relatedness between the isolates was evaluated by cluster analysis with the PFGE patterns. As shown in the dendrogram (Figure 1), serogroup B isolates exhibited a high level of genetic diversity. A total of 40 PFGE patterns were identified in the 52 serogroup B isolates, with the patterns distributed over several distinct clusters. Serogroup B isolates between clusters shared a low level of PFGE pattern similarity, with no single genotype predominated among the isolates. This suggested that the isolates were derived from multiple clonal origins and no single major circulating strain existed. In contrast, the isolates within each of the serogroups A, C, W135, and Y shared high levels of PFGE pattern similarity and PFGE patterns within each of the serogroups tightly clustered (Figure 1). One of the four PFGE patterns (NMEN06.0056) was predominant in serogroup W135 isolates. Of the 31 serogroup W135 isolates typed, 19 were of NMEN06.0056 genotype. Serogroup C clustered tightly with serogroup W135 and shared a 69% pattern similarity.

**Figure 2:**
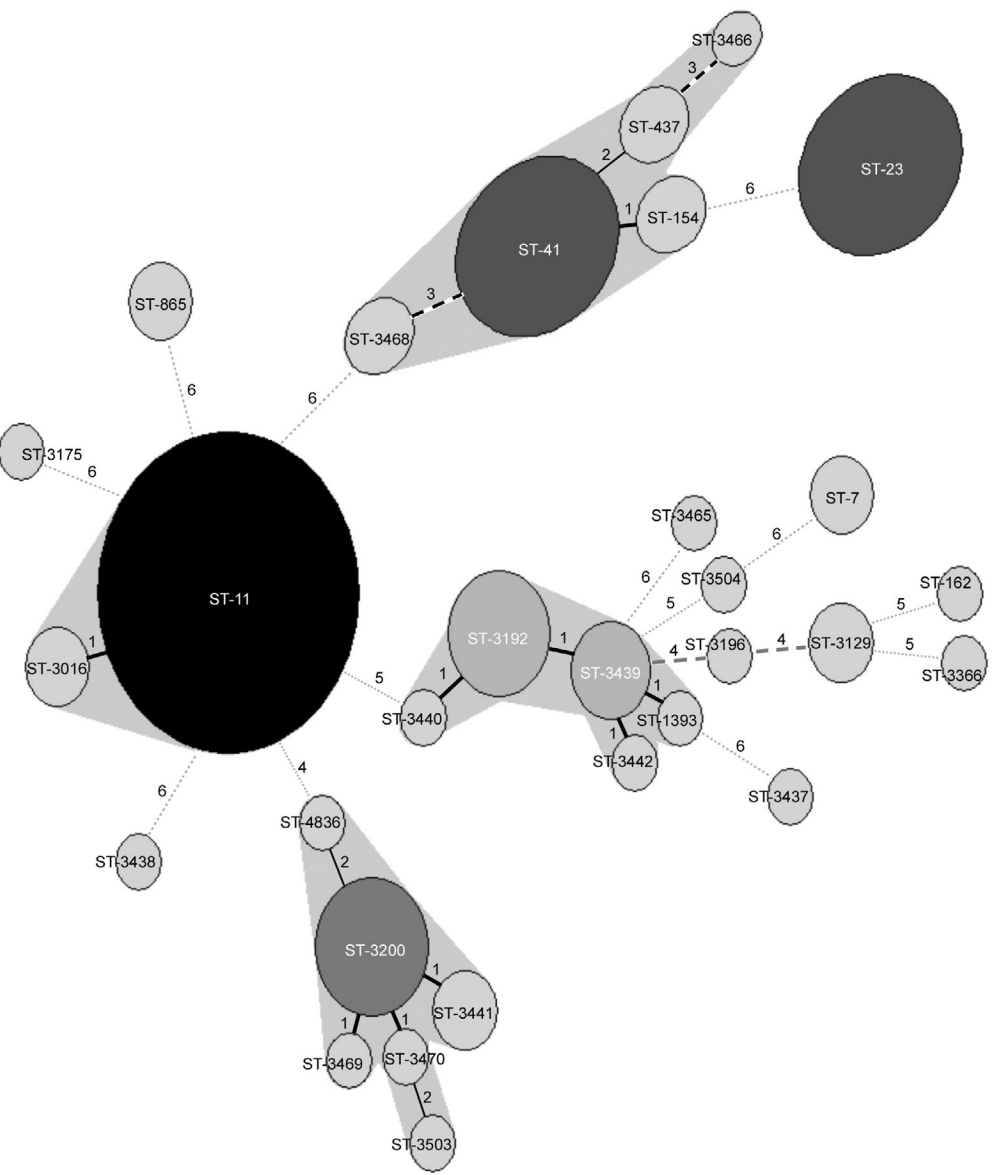
Minimum Spanning Tree diagram consisting of the 30 STs identified. Differences in loci between two STs are numbered. Circle size is proportional to the number of isolates belonging to a ST.

Of the 30 STs, 21 belonged to new ST types, which had not been identified before (Figure 1). Of the 21 new identified ST types, 19 were found in serogroup B isolates. To evaluate the clonal relationships between the isolates, allelic profiles of the 30 STs were analyzed by the MST method. In addition, the STs were deposited into the *Neisseria *MLST database and the relationships with defined clonal complexes were automatically assigned by software in the database. In the MST analysis, STs matching at 4 or more loci were regarded as clonally related. The analysis established the relationship between STs in the clonally related groups, including ST-11 group (ST-11 and ST-3016), ST-41 group (ST-41, ST-154, ST-437, ST3466 and ST-3468), ST-3200 group (ST-3200, ST-3441, ST3469, ST3470, ST-3503 and ST-4836), and ST-3439 group (ST-1393, ST-3192, ST-3442, ST3439 and ST3440) (Figure 2).

Searches of the *Neisseria *MLST database found that some STs belonged to defined clonal complexes, including the ST-7 (ST-5 complex/Subgroup III), ST-11 group (ST-11 complex/ET-37 complex), ST-23 (ST-23 complex/Cluster A3), ST-41 group (ST-41/44 complex/Lineage 3), ST-162 (ST-162 complex), and ST-3465 (ST-32 complex/ET-5 complex) (Figure 1).

In this study, the clonality of the *N. meningitidis *isolates was defined on the basis of ST types. Clonal relationships inferred by the STs were also related to the clustering relationships constructed using the PFGE patterns. Isolates within a clonally related group or clonal complex shared higher levels of PFGE pattern similarity than those in other groups (Figure 1). The serogroup B isolates were assigned to 5 clonally related groups and clonal complexes and 7 unique clones. The ST-41/44 complex/Lineage 3, and the ST-3439 and ST-3200 groups represented 79% of the serogroup B meningococci. The hypervirulent ST-41/44 complex/Lineage 3 consisted of five different STs and the isolates with different STs also shared a lower PFGE pattern similarity. High genetic divergence was also observed in the ST-3439 and ST-3200 groups, containing 5 and 6 STs, respectively. Serogroup A, serogroup W135 (and C), and serogroup Y isolates were characterized as ST-7, ST-11, and ST-23, respectively. They belonged to the hypervirulent ST-5 complex/Subgroup III, the hypervirulent ST-11 complex/ET-37 complex, and the ST-23 complex/Cluster A3, respectively.

## Discussion

Meningococcal disease is a notifiable disease in Taiwan. Physicians and hospital authorities have obligation to report any suspected and confirmed case within 24 hours to local public health department. The disease is infrequently reported in Taiwan. The annual incidence of meningococcal disease was 0.94 cases per 100,000 population in 1953 and then declined to below 0.001 between 1980 and 1987 [16]. In 2001, reported cases dramatically increased with an annual incidence rate of 0.2 cases per 100,000 population. The clinical isolates were limited to serogroups B and W135 in 1996–2000. Thus, the emergence of serogroup A, C, and Y strains in 2001 is of great concern. Our genotyping data suggested that the serogroup A, C, Y and W135 strains originated from hypervirulent lineages that had been circulating in various parts of the world for decades [10,11,17-20]. Even though serogroup W135 meningococci have been detected in Taiwan before 1996, the high level of genetic relatedness between the strains in each of the serogroups A, C, W135, and Y suggested that the original clones have been recently introduced into Taiwan. In contrast, most serogroup B clones could be indigenous or were introduced a long time ago. The high level of genetic diversity among the serogroup B isolates suggested that the meningococci have multiple clonal origins and that strains within a clonally related group are derived from genetic variation during clonal expansion.

The two serogroup A strains were characterized as ST-7, which belong to the hypervirulent ST-5 complex/Subgroup III. ST-5 and ST-7 belonged to the ST-5 complex/Subgroup III and differed only at the *pgm *locus (allele *pgm*3 is characteristic for ST-5 while allele *pgm*19 is for ST-7) [10]. ST-5 strains have been responsible for serious outbreaks in Africa since 1988; however, ST-7 strains are replacing ST-5 strains in Africa since their emergence in 1995 [10]. ST-7 strains are expected to cause the next epidemic wave of serogroup A meningococcal disease in Africa [10,20].

The serogroup W135 meningococcus belonging to ST-11 was responsible for a global outbreak of meningococcal disease in the year 2000 that was related to the Hajj pilgrimage in Saudi Arabia [5,21]. The W135 strain was derived from clonal expansion within the ST-11 complex/ET-37 complex and not from a new clone [11]. Since 2000, serogroup W135 meningococci of ST-11 have been isolated in many African countries, including Algeria, Cameroon, Chad, Senegal, Niger, Central African Republic, and Burkina Faso during the large outbreak of 2001–2 [4,20,22]. Serogroup W135 strains in Taiwan belonged to ST-11; however, they were apparently introduced before the Hajj pilgrimage outbreak. The study by Yazdankhah et al., using a variable-number tandem repeat typing method, also showed that a serogroup W135 isolate from Taiwan in 2000 was genetically distinct from the Hajj-related W135 strain in 2000 [23].

Although serogroup Y strains have not been associated with large epidemics, cases of meningococcal disease caused by this serogroup have been increasing in the USA and Canada since the 1990s [6-8]. Our data indicated that clinical serogroup Y strains recently emerged in Taiwan were derived from a ST-23 clone. ST-23 is also a prevalent type found in Japan [18]. ST-23 strains were the cause of a small-scale outbreak of meningococcal disease in Central Taiwan in 2001. In an unpublished carriage study conducted in 2001, we detected meningococci in 51 (2.56%) of 1988 nasopharyngeal swabs sampled from men of 19–21 years old. Of the isolates, 25 (49%) were serogroup B, 7 (14%) were serogroup W135, 13 (25%) were serogroup Y, and 6 (12%) were non-serogroupable. All the serogroup W135 isolates were characterized as ST-11, and 11 of the 13 serogroup Y isolates were as ST-23. This indicated that, like the ST-11 strains, the ST-23 strains have widely distributed in Taiwan. The new emerged ST-23 strains may have adapted to this environment and then become major circulating strains for meningococcal infection in Taiwan.

The MLST analysis revealed that most serogroup B strains had new STs unique in Taiwan, implying that they could have originated from indigenous clones. In this study we identified three major clonal complex or clonally related groups, namely the ST-41/44 complex/Lineage 3, ST-3200 group and ST-3439 group, which represented 79% of all serogroup B isolates. The ST-3200 and ST-3439 groups probably evolved from indigenous clones. ST-1393, the sole ST of the ST-3439 group that had been identified prior to the study (as shown in Figure 2), was also identified for the first time in a strain from Taiwan. In contrast, the strains within the hypervirulent ST-41/44 complex/Lineage 3 most likely were derived from an imported clone. This is an example of an imported clone that has successfully adapted to a new environment and then become a major circulating causative agent. The ST-11 and ST-23 strains have also successfully adapted within the Taiwanese population and continue to cause disease throughout 2003–2004.

## Conclusion

Serogroup B isolates collected in 1996–2002 were derived from multiple clonal lineages. Since most of the isolates were characterized as new STs unique to Taiwan, they should derive from indigenous clones. Although three major clonal lineages were defined by phylogenetic analysis of the allelic profiles of the STs, no a predominant PFGE:ST genotype emerged at a particular time. Serogroup B strains caused only sporadic infections and were never responsible for any apparent disease outbreak in Taiwan.

The emergence of serogroup A, C and Y strains contributed partly to the dramatic increase in cases of meningococcal disease in 2001–2002. In our record, serogroup A, C and Y strains emerged for the first time in 2001. Serogroup Y strains had apparently caused a small meningococcal disease outbreak in central Taiwan in 2001. Strains of serogroups A, C and Y belonged to three worldwide epidemic clones within the ST-5 complex/Subgroup III, ST-11 complex/ET-37 complex, and ST-23 complex/Cluster A3. Introduction of hypervirulent *N. meningitidis *strains into Taiwan increases the burden of the disease in the country since new strains may adapt to the environment similar to the serogroup W135 strains, which have become major circulating strains in Taiwan.

## Competing interests

The author(s) declare that they have no competing interests.

## Authors' contributions

CS Chiou initiated and managed the project, analyzed data, and wrote the report. JC Liao did most of the PFGE and all of the MLST work and TL Liao did part of the PFGE analysis. CC Li was in charge of the Bionumerics database and clustering analyses. CY Chou, HL Chang, SM Yao and YS Lee were in charge of the *N. meningitidis *strain serotyping and strain collection in the four Taiwan CDC laboratories. All authors read and approved the final manuscript.

## Pre-publication history

The pre-publication history for this paper can be accessed here:


